# Revealing the complete mitogenome sequence of *Hypsugo alaschanicus* based on next generation sequencing

**DOI:** 10.1080/23802359.2017.1372705

**Published:** 2017-08-30

**Authors:** Yanshuang Shi, Shuai Zhao, Xiaomin Han, Chunzhu Xu

**Affiliations:** College of Life Science, Northeast Agricultural University, Harbin, China

**Keywords:** *Hypsugo alaschanicus*, mitochondrial genome, next generation sequencing, phylogenetic analysis

## Abstract

*Hypsugo alaschanicus* belong to Chiroptera, which is the only type of mammals with the real ability to fly. The complete mitochondrial genome of *H. alaschanicus* based on next generation sequencing data thus determined had 37 genes for 13 proteins, 22 tRNAs, and 2 rRNAs together with a major non-coding region in a typical gene arrangement of vertebrate mitogenomes. Phylogenetic analysis shows that Pipistrellini is multiline origin, *Pipistrellus*-like bats can be divided into three groups: Pipistrellini-Nyctalini, Vespertilionini-Eptesicini, and Asian Pipistrelles. *Hypsugo* alone become a clade, *Vespertilio* and *Eptesicus* phylogenetic relationship are closer, *Pipistrellus* and *Nyctalus* have a close phylogenetic relationship.

*Hypsugo alaschanicus* of the genus *Hypsugo* are member of the Chiroptera family Vespertilionidae, the only type of mammals with the real ability to fly, there are more than 1200 species in the 20 families (Gunnell and Simmons 2005; Lei and Dong 2016). *H. alaschanicus* mainly regard insects as food, its faeces as a Chinese herbal medicine have the effect of removing heat to brighten vision. And in agricultural production, the excreta is a rare agricultural fertilizer (Schnitzler and Kalko 2001). The sequencing of the mitochondrial genome of *H. alaschanicus* from Korea had been completed through Sanger sequencing (Kim and Park 2015). In this study, *H. alaschanicus* from China was sequenced by next-generation sequencing. The use of different sequencing methods for different areas of *H. alaschanicus* are compared in order to obtain the difference of composition of *H. alaschanicus* mitochondrial genome from two regions.

*H. alaschanicus* was collected from China (45°44′41.14″N, 126°43′37.66″E), and stored in the animal specimen room of Northeast Agricultural University with an accession number NEAU-2016-H12181. The mitochondrial genome was extracted by the phenol–chloroform method, and then dissolved in TE buffer (10 mmol Tris, 1 mmol EDTA pH 8.0) and kept at −20 °C. The whole genome shotgun method was used to construct the library, and next-generation sequencing technique was used to sequencing based on the Illumina MiSeq sequencing platform. The complete mitogenome of *H. alaschanicus* was 17,039 bp in length, a total of 37 genes (13 protein coding genes, two ribosomal RNAs, and 22 transfer RNAs) and one control region (GenBank accession number MF459671). Its overall base composition was 33.89% (A), 22.72% (C), 12.86% (G), 30.53% (T), and G + C content 35.58%. One gene (ND6), and eight tRNAs were encoded on the J-strand, while the remaining 12 PCGs, 14 tRNAs and both rRNAs were encoded on the N-strand. In contrast, the length of the *H. alaschanicus* mitochondrial genome collected from Korea was 17,300 bp with the nucleotide composition of 34.1% (A), 22.4% (C), 12.6% (G), and 30.9% (T). It can be seen that in terms of mitochondrial genome composition there are no significant difference between Korea and China.

This study use the MrBayes v3.2 (Ronquist et al. 2012) software and MEGA6 (Tamura et al. 2013) based on 13 mitochondrial PCGs of 17 species build phylogenetic trees to study its phylogenetic relationship and set the *Equus caballus* as an outgroup. The result (Figure 1) shows that *Murina* and *Myotisis* have a close phylogenetic relationship and co-locate on a large clade; *Eptesicus*, *Vespertilio*, *Hypsugo, Pipistrellus,* and *Nyctalus* have a close phylogenetic relationship which co-locate on an another large clade. The above results are similar to the previous studies (Volleth and Heller 1994; Kawai et al. 2002; Hoofer and Van Den Bussche 2003). Phylogenetic analysis shows that the Pipistrellini is multiline origin, *Pipistrellus*-like bats can be divided into three groups: Pipistrellini-Nyctalini, Vespertilionini-Eptesicini, and Asian Pipistrelles. *Hypsugo* alone become a clade, *Vespertilio* and *Eptesicus* phylogenetic relationship are closer, *Pipistrellus* and *Nyctalus* have a close phylogenetic relationship.

**Figure 1.**
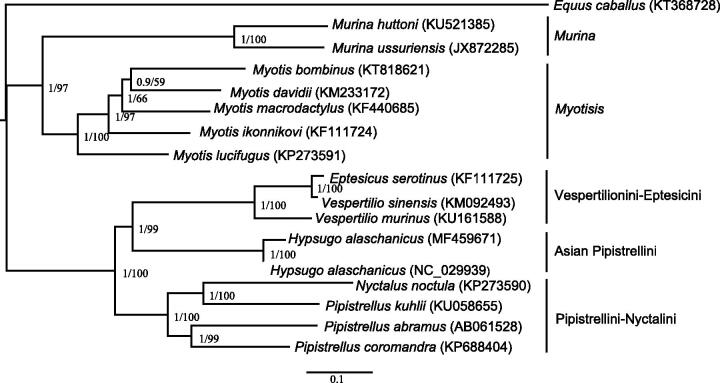
Phylogenetic tree obtained from maximum likelihood (ML) analysis and mrbayes(BI) anglysis based on 13PCGs. BI posterior probabilities/ML bootstrap value are indicated at internal nodes.
